# Nanoparticles in the New Era of Cardiovascular Therapeutics: Challenges and Opportunities

**DOI:** 10.3390/ijms24065205

**Published:** 2023-03-08

**Authors:** Pingping Yang, Jun Ren, Lifang Yang

**Affiliations:** 1 Department of Anesthesiology, Xi’an Children Hospital, Xi’an 710032, China; 2 Department of Cardiology, Shanghai Institute of Cardiovascular Diseases, Zhongshan Hospital Fudan University, Shanghai 200032, China; 3 Department of Laboratory Medicine and Pathology, University of Washington, Seattle, WA 98195, USA; 4 Key Laboratory of Precision Medicine to Pediatric Diseases of Shaanxi Province, Xi’an 710003, China

**Keywords:** cardiovascular disease, nanoparticle, therapy, ischemic heart disease, ischemia-reperfusion injury

## Abstract

Cardiovascular disease (CVD) is the leading cause of morbidity and mortality worldwide. Although a cadre of therapeutic strategies have been made available for CVDs in the clinical setting, predominantly through medication and surgery, these do not fully address the clinical needs of patients with CVD. As a new technique for CVD treatment, nanocarriers are employed to modify and package medications to ease the targeting of tissues, cells and molecules within the cardiovascular system. Nanocarriers are made of biomaterials, metals, or a combination of these materials, with sizes similar to bioactive molecules such as proteins and DNA. Cardiovascular nanomedicine (CVN) has only surfaced in recent years and is still in its infancy. Ample studies have displayed promise for the clinical utility of nanomedicine techniques, courtesy of continued perfection in nanocarrier design to optimize drug delivery and treatment outcomes. Here in this review, we will summarize the research advances in the literature on nanoparticles in the management of CVDs, including ischemic and coronary heart disease (e.g., atherosclerosis, angina pectoris and myocardial infarction), myocardial ischemia-reperfusion injury, aortic aneurysm, myocarditis, hypertension, and pulmonary artery hypertension and thrombosis.

## 1. Introduction

Cardiovascular disease (CVD) remains the leading cause of death worldwide. With continuous advances in diagnostic and surgical techniques, as well as increased awareness of disease and lifestyle, CVD mortality has been declining, although it still remains high with approximately 18.6 million dying from CVD worldwide in 2020, a 18.7% rise from 2010 [1,2]. Consequently, CVD imposes a huge burden on health care and the global economy. Although ample therapeutic strategies have become available for CVD, including anticoagulant drugs, antiplatelet drugs, thrombolytic drugs and antilipidemic drugs, as well as procedures for vascular bypass grafting and stent implantation [3,4], the clinical demand still cannot be fully met. For instance, traditional dosage forms of medications usually suffer from severe pitfalls including poor absorption, short plasma half-life, high plasma clearance, toxic side effects and low efficacy [5].

Nanomedicine is the application of nanotechnology in monitoring, diagnosing, preventing, repairing or curing diseases and damaged tissues in biological systems [6]. This term was first coined in the book “Unbounding the Future: The Nanotechnology Revolution”, published in 1991 [7]. Nanoparticles typically range between 1 and 100 nm in size, similar to large molecules such as proteins and DNA. Nanocarriers can be generated from biomaterials (e.g., lipids, peptides), metals or combinations of these materials. The surface of a nanoparticle can be modified with peptides, polymers or antibodies to best facilitate the delivery to specific targets in cells or tissues, thereby increasing drug aggregation and retention at specific sites to avoid systemic side effects [8]. 

**Figure 1:**
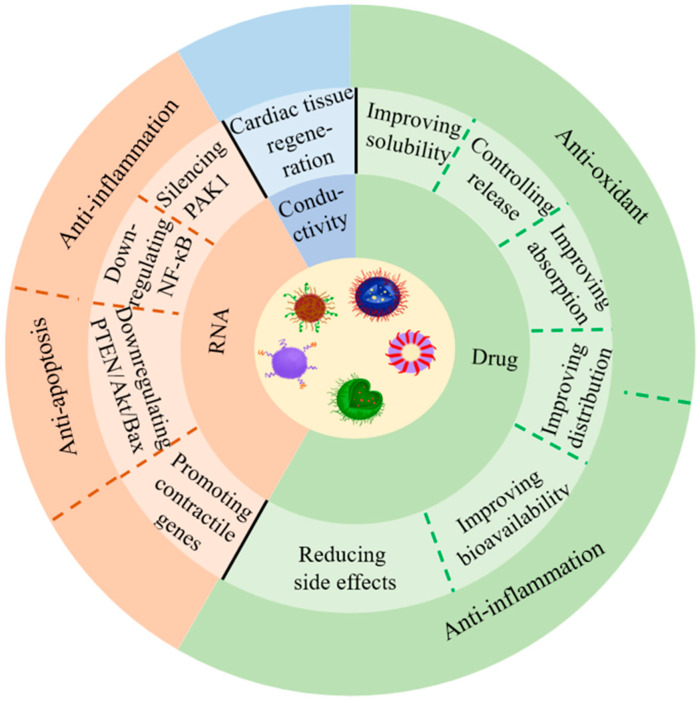
Application of nanoparticles for the treatment of CVDs.

Although nanomedicine techniques are only beginning to be recognized in cardiovascular therapy, many studies have already generated some promising results, with mainstream research focusing on the design of nanocarriers to improve drug delivery and thereby treatment outcomes [9,10,11,12,13,14]. Several studies have demonstrated that delivering drugs to heart tissues, cells, and molecules through nanocarriers can significantly increase oral availability and half-life, reduce side effects, and optimize therapeutic outcomes [15,16,17,18,19]. Besides, nanoparticles can also control the drug release modality [20,21], such as drug release conditions (acid–base situation) [22], velocity [23], and duration [24,25]. As early as the mid-2000s, scientists, including our own group, designed polymorphic nanocarriers to carry free DNA for gene therapy through transfection [26,27]. Nanoparticles have been demonstrated to be vital in cardiac regeneration, in addition to being an effective means of drug and nucleic acid delivery [28]. Moreover, recent research has shown that nanoparticle-gated electrokinetic membrane sensors can also detect the level of PON1 in HDL in order to evaluate the risk of CVDs [29]. Herein, we summarize and review the research advances in the literature on nanoparticles in the therapy of CVDs, including ischemic heart disease (e.g., atherosclerosis (AS), angina pectoris, myocardial infarction (MI)), myocardial ischemia-reperfusion injury (IRI), aortic aneurysm, myocarditis, hypertension, and pulmonary artery hypertension (PAH) and thrombosis. In addition, we also discuss the challenges and opportunities of nanoparticles in the new era of CVD therapeutics. Figure 1 illustrates the application of nanoparticles in the treatment of CVDs.

## 2. Nanoparticles for the Treatment of CVD

Nanocarriers carry therapeutic drugs of interest and deliver them directly to the specific sites through passive or active targeting. In passive targeting, nanodrugs reach the specific site through highly selective permeability and penetration. For active targeting, nanodrugs bind to site-specific molecules or cell-specific targeting ligands [5]. Ample efforts have been engaged towards active targeting nanoparticles in the treatment of CVDs [30,31]. Nanoparticles are found to target not only specific cells, such as endothelial cells, monocyte/macrophages, and platelets, but also ligands, including specific antibodies (e.g., vascular cell adhesion molecule-1 antibody [32]), peptides (e.g., mitochondrial targeting peptide [33]), and aptamers. Furthermore, dual targeting ligands can be applied to different cells [34] and different receptors on the same cell surface [35] to improve the efficiency of drug delivery. Several researchers have noted a role for extracellular matrix (ECM) components as possible new targets [36]. In general, the principle of rational design for a successful nanomedicine in the treatment of CVDs is to adopt nontoxic biological, metal or biomimetic materials as carriers, loaded with drugs of interest or RNA sequence and modified with relevant ligands to target specific sites, cells, or molecules. Ultimately, optimized velocity, duration and other conditions may be achieved for drug release.

In addition to applications in CVD therapeutics, nanoparticles can also be employed for imaging and diagnostic purposes. Not only can nanoparticles actively target macrophages, endothelial cells, vascular smooth muscle cells (SMCs), activated platelets, and fibrin [37], they can also be utilized for cardiovascular imaging by actively targeting ligands (e.g., vascular cell adhesion molecule-1 [38]), and probes, which could even detect infarcted myocardium and ROS levels [39]. In particular, nanoparticles are capable of improving the sensitivity of nuclear imaging, MRI, ultrasound, fluorescence imaging, and multimodal imaging to diagnose CVDs [40], fostering their applications in thrombosis and ischemia imaging, blood pool imaging, angiogenesis imaging, and stem cell imaging [41].

### 2.1. Nanoparticles for Ischemic Heart Disease

Ischemic heart disease is the most common form of heart disease and the leading cause of disability and mortality worldwide. It can be divided into four types: stable angina, unstable angina, MI and sudden cardiac death [42]. The different approaches based on nanoparticles for the treatment of ischemic heart disease are shown in Table 1.

#### 2.1.1. Nanoparticles for Atherosclerosis (AS)

**Figure 2:**
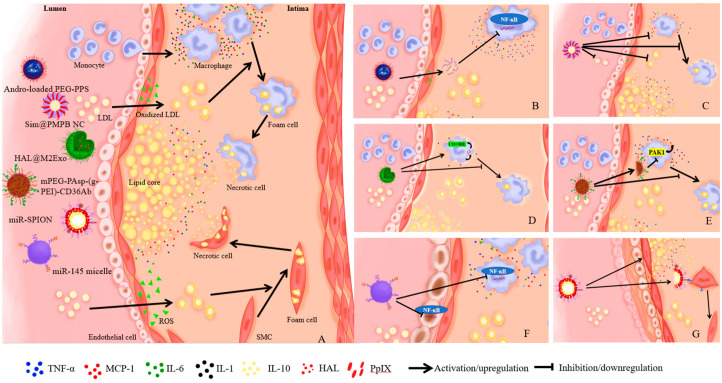
The mechanism of nanomedicines of different strategies for AS treatment. (**A**) shows the pathological processes of AS. (**B**) shows that Andro-loaded PEG-PPS could scavenge excess ROS, inhibit the expression of pro-inflammatory cytokines IL-6 and MCP-1, as well as block NF-κB signaling pathway, to dampen inflammation [18]. (**C**) shows that Sim@PMPB NC could scavenge excess ROS, inhibit expression of pro-inflammatory cytokines (IL-6, MCP-1 and TNF-α), reduce LDL, inhibit oxidized LDL internalization and foam cell formation [43]. (**D**) shows that HAL@M2exo could secrete anti-inflammatory cytokines IL-1 and IL-10, increase anti-inflammatory capacity by CO and bilirubin, inhibit oxidized LDL internalization and foam cell formation [46]. (**E**) shows that mPEG-PAsp-(g-PEI)-CD36Ab could reduce CD36 expression by target macrophages, inhibit upregulation of IL-6 and MCP-1 by silencing PAK1 gene, and decrease the formation of foam cells [48]. (**F**) shows that miR-SPION could downregulate NF-κB signaling pathway both in macrophages and endothelial cells, and further reduce inflammation [50]. (**G**) shows that miR-145 micelle could target the CCR2 expressing in vascular SMCs, promote the transition of SMCs from synthetic phenotype to contractile phenotype, and reduce the size of plaque lesions and necrotic core area [51].

AS is a disease in which sclerotic plaques form due to excessive accumulation of low-density lipids in arterial intima, and is considered the most common cause of myocardial ischemia and infarction [3,55]. The main trigger of this chronic disease is excessive cholesterol levels in the blood, leading to changes in arterial endothelial permeability, to favor the migration of lipids from the blood stream to the arterial wall [56]. The diagnosis is primarily based on a variety of invasive and non-invasive tests to evaluate the number and position of atherosclerotic plaques or blockades in patients with ischemic signs and symptoms, depending on the organ involved. Classical pharmacological interventions are the most widely used strategies, including lipid regulation, anti-platelet aggregation, and inhibition of thrombosis. However, these maneuvers often suffer from pitfalls including low location specificity, high volume of drug administration, and side effects. Inflammation and oxidative stress are the two crucial factors in the pathogenesis of AS, making targeted therapy a possible new strategy in AS management [39,43]. Nanodrugs were found to prolong the half-life of drug dissolution, reduce toxic side effects and improve their distribution. Zhong and associates developed a plug-and-play nanoplatform with targeted drug delivery and drug release controlled by acid, which displayed pronounced therapeutic benefits in AS [22]. In a preclinical in vivo model, ApoA1 PA nanofibers with the liver X receptor agonist GW3965 (LXR) were employed for safe and targeted delivery to areas of atherosclerotic plaque to reverse AS [57]. Therefore, we believe that nanoparticles can improve the delivery of drugs to the lesion site, and enrich drug concentration in the lesion site, ultimately benefiting AS treatment. Figure 2 displays the perceived mechanism of nanomedicines of different strategies for the treatment of AS.

(1) Anti-oxidant strategy

Vulnerable plaques are localized in a ROS-rich microenvironment, and excessive production of reactive oxygen species (ROS) can evoke oxidative damage to lipids and proteins, further provoking apoptosis and necrosis. Moreover, as a second messenger in cell signaling, excess ROS activates NF-κB signaling to promote the production of pro-inflammatory cytokines, making antioxidant therapy an effective and promising strategy to mitigate the development of AS [58].

Andrographolide exhibits excellent anti-inflammatory properties by blocking NF-κB signaling pathway, although its poor water solubility has greatly restricted its application in vivo. Polymeric micelle, loaded with andrographolide, assembled from an amphiphilic dimer copolymer poly(ethylene glycol)-poly(propylene sulphide) (PEG-PPS) with oxidative sensitivity, could effectively inhibit pro-inflammatory cytokines IL-6 and MCP-1 and alleviate oxidative stress in macrophages. In addition, micelle carriers were able to reduce oxidative stress by reacting with ROS reaction in aorta. Therefore, micelles possess great therapeutic potential with relatively low side effects in AS treatment [18] (Figure 2B). A biocompatible simvastatin (Sim)-loaded nanocube based on porous manganese-substituted prussian blue (PMPB) analogues (Sim@PMPB NC) has been demonstrated to scavenge excess ROS, eliminate oxidative stress, as well as inhibit AS plaque macrophage infiltration, proinflammatory cytokines (IL-6, MCP-1, TNF-α) secretion, intravascular SMC proliferation and matrix metalloproteinase-9 (MMP-9) expression. It also inhibits the internalization of oxidized LDL and the formation of foam cells. Therefore, Sim@PMPB NC should hold great potential for clinical translation for AS treatment given its ability in stabilizing atherosclerotic plaques and alleviating AS progression [43] (Figure 2C). For example, a biomimetic drug delivery system was derived from ROS-responsive nanoparticles encapsulated by macrophage membranes, not only to avoid the clearance of nanoparticles by the reticuloendothelial system but also to guide ROS-responsive nanoparticles to inflammatory tissues to achieve specific release, in addition to the macrophage membranes, by isolating pro-inflammatory cytokines to suppress local inflammation. This co-operative effect of drug treatment and inflammatory cytokine isolation by the bionic delivery system improves the therapeutic efficacy of AS treatment [44]. TPCD NP, a broad-spectrum ROS eliminating material synthesized by a superoxide dismutase mimetic reagent Tempol (a hydrogen peroxide-eliminating compound of phenylboronic acid pinacol ester and a cyclic polysaccharide β-cyclodextrin) was found to reduce systemic and local oxidative stress, internalization of oxidized low-density lipoprotein and inflammatory cell infiltration in plaques. TPCD NP by intravenous injection could accumulate in atherosclerotic plaques and effectively delay the development of AS in atherosclerotic lesions of apolipoprotein E-deficient (ApoE-/-) mice by passive targeting. Compared to controls, TPCD NP treatment produced more stable plaque development, fewer cholesterol crystals, smaller necrotic nuclei, thicker fibrous caps, and fewer macrophages [45]. Therefore, nanoparticles can be employed to target the plaque region of AS to alleviate oxidative stress, inhibit the internalization of oxidized LDL and the formation of foam cells, ultimately effectively retarding AS pathogenesis.

(2) Anti-inflammation strategy

Persistent inflammation in the atherosclerotic plaque is a key driving force for rupture of unstable atherosclerotic plaques. Therapeutic strategies to reduce the pro-inflammatory response may help to stabilize high-risk plaques. Dexamethasone (Dex) is a long-acting synthetic glucocorticoid that inhibits proinflammatory responsiveness. The main effect of Dex is to inhibit the expression of pro-inflammatory cytokines (IL-1, IL-6, IL-8 and TNF-α), involved in the migration of leukocytes to extravascular space, and cell adhesion molecules. Owing to the absence of natural affinity for endothelial cells, Dex may lead to side effects such as hypertension, osteoporosis, and hyperglycemia during treatment of vascular diseases. A synthesized nanodrug, Ab-NG-DEX, designed by Eckmann and team, could be targeted to endothelium and specifically aggregate at the lesion site via conjugation with an antibody (Ab) directed to Intercellular Adhesion Molecule-1 [59]. The nanocarrier lysozyme dextran nanogels (NG) has rapid carrier release and low cytotoxicity. Xie and associates designed M2 phenotypic macrophage-derived exosomes (HAL@M2exo) and found a 75.2% reduction in aortic lesion size in mice following treatment with HAL@M2Exo compared with controls [46]. These authors demonstrated that HAL@M2 Exo not only targets and alleviates atherosclerotic inflammation through surface chemokine receptors and secretion of anti-inflammatory cytokines, but also utilizes CO and bilirubin, which are produced during hemoglobin synthesis from the natural precursor hexyl 5aminolevulinate hydrochloride (HAL), to increase anti-inflammatory capacity and improve therapeutic efficacy. In addition, the intermediate protoporphyrin IX (PpIX) can be used for fluorescence imaging and tracking of AS during HAL-mediated heme biosynthesis (Figure 2D). Wang and associates reported that biomimetic nanoparticles MM/RAPNPs coated with macrophage membrane loaded with rapamycin effectively inhibited the phagocytosis of macrophages [47]. MM/RAPNPs significantly delayed the progression of AS after four weeks of treatment via targeting and aggregating at lesion sites of atherosclerotic mice in vivo, suggesting that biomimetic nanoparticles may be a safe, effective, and promising drug delivery system for the treatment of AS. A CD36 antibody-modified siRNA, based on the mPEG-PAsp-(g-PEI) vector could reduce CD36 expression by targeting macrophages, inhibit the upregulation of IL-6 and MCP-1 by silencing PAK1 gene, decrease the formation of foam cells, and alleviate AS pathogenesis [48] (Figure 2E). Intravenous mixed nanostructures can alleviate local and systemic inflammation of immune cells. The 100-nanometer spherical polymer nanostructure (SPNs), loaded by methotrexate (MTX), is specifically absorbed by macrophages and rapidly releases MTX to reduce the production of proinflammatory cytokines and inflammation, an effective strategy to retard the progression of AS [23]. These findings convincingly suggest that nanoparticles can be modified by antibodies targeting endothelial cells and macrophages, or by macrophage exosomes and biomimetic techniques to downregulate pro-inflammatory cytokines and promote the secretion of anti-inflammatory cytokines. Moreover, nanoparticles can be used to deliver anti-inflammatory drugs to the lesion site through active targeting, thus alleviating inflammation to halt AS progression.

(3) Other strategies

In addition to antioxidant and anti-inflammatory strategies, other therapeutic options also prevail for AS. Maiseyeu and colleagues observed the regulatory effect on vascular targets from the nanoparticle GlpNP, loaded with an agonist of glucagon-stimulated 1 receptor liraglupeptide, particularly in the case of SMC inflammation [49]. Both in vitro and in vivo (including lineage tracing) studies exhibited that 1 μg/kg dose of GlpNP retarded AS, independent of pancreas or central nervous regulation. Gene therapy for regulation of AS is gaining rising attention. Assembling therapeutic oligonucleotides into three-dimensional spherical nucleic acid nanostructures can improve systemic delivery for plaque and AS treatment. These non-cationic nanoparticles contain a shell of microRNA-146a oligonucleotides that downregulate the NF-κB signaling pathway, for achieving transfection-free cellular entry into macrophages and endothelial cells. It also reveals the potential of nucleic acid nanotechnology to treat CVDs [50] (Figure 2F). Furthermore, one study from the United States reported that miR-145 micelle targeted the C–C chemokine receptor-2 (CCR2) expressing in vascular SMCs, promoted the transition of SMCs from synthetic phenotype to contractile phenotype, and reduced the size of plaque lesions and necrotic core areas in early and middle atherosclerotic mice. These effects occur with preserved collagen structure. It is suggested that miR-145 micelles may play an important role in alleviating AS and in its long-term therapeutic potential as a chronic condition, in view of their targeting and therapeutic efficacy and biocompatibility in vivo [51] (Figure 2G). Given the oral targeting effect of BIN/YCs derived from a yeast-derived microcapsule (YC), BIN/YCs exhibited a synergistic effect between the modulation of plaque lipid levels and inhibition of plaque inflammation. At low doses Bindarit (BIN) significantly reduced the formation of atherosclerotic plaque and successfully achieved a preventive effect on AS at feasible low dosage for chronic disease [52]. Shang and coworkers designed a bifunctional supramolecular nanofiber that combines the biological activity of insulin-like growth factor-1 (IGF-1) and the anti-inflammatory properties of naproxen (Npx) to effectively inhibit AS development via regulating cholesterol efflux and inflammation, providing a promising nanomedicine for the treatment of AS [53]. In addition, a novel macrophage-targeted nanoparticle, with a combination of rapamycin and IL-1 receptor antagonists, is successfully constructed and developed for AS treatment, suggesting the clinical utility of metal-organic framework-based immunomodulatory nanoplatforms in atherosclerotic CVD or other inflammatory diseases [60]. To this end, nanoparticles not only play an important role in gene regulation in AS treatment, but also possess potential to prevent AS progression though oral, as opposed to intravenous, administration.

#### 2.1.2. Nanoparticles for Angina Pectoris

Angina pectoris is a serious medical condition with insufficient blood supply to meet the myocardial oxygen demand. When angina occurs, patients may experience severe pain in the chest, shoulders, neck, arms and back due to insufficient blood supply to the heart muscle caused by blockage or narrowing of the arteries [61,62]. Based on the typical attack characteristics, signs, and relief with nitroglycerin, in combination with the existence of coronary susceptibility factors, coronary angiography results, and exclusion of other causative factors, angina pectoris can generally be diagnosed. Classical drugs for stable angina pectoris include nitrate, β-blocker, calcium channel blocker and antilipemic drugs, although many of these medications display a poor bioavailability or efficacy [5]. Intriguingly, nanotechnology has unique advantages in improving pharmacokinetics. It has been reported that the oral bioavailability and half-life of Ivabradine can be increased by adding it into polymeric nanoparticles [15]. Another study also reported a significant increase in the uptake of verapamil by the combination of verapamil and dextran in nanoliposome [63]. In addition, a novel biocomposite polymeric nanofiber for sublingual delivery of nicorandil can reduce mucosal ulceration and improve drug bioavailability successfully [16]. Zhuge and team studied the targeting effect of drug-loaded liposomes on cardiac radiofrequency ablation (CA) and found that the concentration of amiodarone hydrochloride (ADHC) was elevated by a 4.1-fold increase in the heart after 20 min [64]. Moreover, nanocarriers can be used in combination to improve therapeutic efficacy and reduce the undesired side effects. For instance, RGD-S/P-LPNs can improve the therapeutic efficacy by combination of salvianolic acid B (Sal B) and panax notoginsenoside (PNS) in acute myocardial ischemia [65]. To sum up, nanotechnology can improve the absorption and distribution of drugs in the body by carrying one or more drugs, thus enhancing the therapeutic effect.

#### 2.1.3. Nanoparticles for Acute Myocardial Infarction (AMI)

Myocardial infarction (MI) is characterized by extremely high morbidity, disability, mortality, and recurrent morbidity. In the absence of adequate oxygenation of the coronary artery, myocardial cells become ischemic or even necrotic. When MI occurs, myocardial tissues release large amounts of cytokines, leukocytes, TNF-α, IL-1, and IL-6, and the amount produced correlates positively with the degree of myocardial damage and the death of the myocardium, thus the reduction of these injury factors has a therapeutic effect [5]. The diagnosis of AMI can be made by combining typical clinical manifestations, characteristic changes in the electrocardiography, and hallmarks of myocardial injury such as troponin-C and LDH. AMI is the result of epicardial coronary artery occlusion, and percutaneous coronary intervention (PCI) and thrombolysis are the standard clinical treatments to restore blood flow to the ischemic area [66]. There are many problems with the current pharmacological treatment of MI. First of all, most drugs are targeted with limited access to treatment of the ischemic myocardium. Secondly, therapeutic drugs do not reach the target site in sufficient quantities, and the majority of the drugs do not remain at the infarct boundary or become inactive for a short time, related to the drug delivery technique, drug half-life and washout mechanism of the contracting myocardium [67]. The delivery of drugs via nanoparticles is a new therapeutic strategy. Torrieri and coworkers designed an acetalated dextran-based nanosystem decorated with two different peptides, linTT1 and atrial natriuretic peptide (ANP), which target macrophages and cardiac cells associated to the atherosclerotic plaques, respectively, and the system has a potential ability to exploit the “hitchhike” effect on M2–like macrophages and to improve the ability of ANP peptide to target the infarcted heart in a dual targeting strategy [34]. Therefore, nanoparticles can solve the pitfalls of traditional drugs by dual targeting ligands to improve AMI treatment. 

Apoptosis, inflammation, and fibrosis are three important factors in pathogenesis of MI, the targeting of which has been shown to ameliorate MI and rescue cardiac function. Xue and associates designed macrophage membrane coated nanoparticles (MMNPs) containing miR199a-3p, and they found MMNPmiR199a-3p could suppress hypoxia-induced apoptosis, inflammation and cardiac fibrosis, and promote cell proliferation in MI mice by binding to TNF-α, IL-1β, IL-6 through receptors, thus ameliorating left ventricular remodeling and cardiac functions, and protecting against MI [54]. Pirfenidone (PFD) is one of the most commonly used anti-fibrotic drugs, however, its clinical use is limited. Perfluoropentane-Pirfenidone@Nanodroplets-Polyethylene glycol 2000 (PFP-PFD@NDs-PEG) has been successfully synthesized, which has a great potential in the treatment of MI, and by loading it into acellular peritoneal matrix (APM) the total time of PFD release was increased, which can achieve sustained release and have good therapeutic effect on myocardial fibrosis in MI rats [24]. In addition, PFD inhibits the conversion of cardiac fibroblasts to cardiac myofibroblasts and reduces the synthesis and secretion of type I and type III collagen by cardiac myofibroblasts. Tel-doped supramolecular nanofibers (TDCNfs) have a significant protective effect on injured myocardium due to their good targeting and downstream pathway, having superior potential in combating adverse cardiac outcomes following MI by reducing apoptosis and inflammation, enhancing antifibrotic potential and limiting toxicity [35]. Curcumin possesses a therapeutic effect against heart disease, while heart targeted peptide-extracellular vesicles-curcumin (CTP-EVs-CUR) improves its bioavailability to achieve a good cardioprotective efficiency. Combined utilization of curcumin and miR-144-3p, a major contributor in curcumin-mediated therapeutic effects by downregulating the PTEN/Akt/Bax signaling pathway involved in apoptosis, could enhance the heart-targeting ability and cardioprotective effect [19]. Moreover, in the case of arrhythmias after MI, scientists have combined thermal and conductive graphene quantum dots (GQDs) with polyethylene glycol (PEG), which increases the solubility, efficiency, and bioavailability of GQDs, to normalize abnormal electrocardiograms in rats after MI [68]. However, there is no evidence for the utility of nanoparticles for other causes of arrhythmias. In conclusion, nanoparticles may target infarct regions through carrying drugs or RNA and by selectively employing ligand modifications, therefore improving drug bioavailability and therapeutic efficacy of drugs in myocardium, opening a new avenue for the treatment of MI.

### 2.2. Nanoparticles for Myocardial IRI

Blood reperfusion can not only recover and rescue tissue from hypoxia, but also cause severe injury via oxidative stress, a process commonly known as IRI [69]. It is noteworthy that attention to IRI may affect the optimal timing of reperfusion, and it is the most effective treatment for ischemic heart diseases, such as AMI [70]. ROS increases after reperfusion, stimulates endothelial cells, causes the infiltration of neutrophils, and further aggravates oxidative stress. The new evidence indicates that the main cause of myocardial IRI is primarily attributable to the high production of ROS following the recovery of ischemic myocardial blood flow [71], and that excessive production of ROS dramatically increases the death rate of patients [72]. Exenatide pretreatment can reduce oxidative stress and enhance the activity of mitochondrial ATPase, however, it has short half-life. Nonetheless, the pretreatment of exenatide-loaded poly (L-lysine) -poly (ethglycol) poly (L-lysine) (PLL-PEG-PLL) nanoparticles can reduce myocardial IRI by reducing LVEDP and HR, and improving LVEF, dp/dtmax and BP. Moreover, PLL-PEG-PLL as a carrier can prolong the half-life of exenatide in circulation and further enhance the effect [73]. To this end, alleviation of ROS levels to combat oxidative stress is key to treating IRI, and nanoparticles can help to offset shortcomings of conventional drugs to enhance efficacy.

It is noteworthy that ROS-responsive fluorescent nanoprobes can also evaluate myocardial IRI function. In a mouse model of MI, fluorescent nanoprobes were highly specific to ischemia-reperfusion myocardium within the first 24 h after reperfusion. Therefore, these nanoparticles have great potential and feasibility in detecting infarcted myocardium and measuring ROS levels, and are specific for infarcted myocardium [39]. Ginsenoside Rg3 (Rg3) was shown to exhibit excellent antioxidant activity. Li and team successfully synthesized poly (ethylene glycol)-poly (propylene sulfide) (PEG-b-PPS), a ROS-sensitive nanoparticle for the delivery of Rg3 [74]. The release of PEG-b-PPS-Rg3 to Rg3 depends on ROS and provokes antioxidant responses through interaction with FoxO3a, resulting in inhibition of oxidative stress, inflammation and fibrosis. More importantly, PEG-b-PPS-Rg3 overtly improved survival following myocardial IRI in rats compared to the free Rg3 group. These findings denote the unique property of PEG-b-PPS-Rg3 being sensitive to cardiac ROS production, proving a potent ROS clearance capacity. Cheng and team established a dual-shell polymer nanoplatform, a multistage continuous targeted drug delivery carrier (designated as MCTD-NPs), consisting of ischemic myocardial-targeting peptide STSMLKA, mitochondrial-targeted ligand SS-31 and ROS scavenger resveratrol (RSV), in order to achieve accurate administration and better clearance of excess ROS after myocardial IRI [33]. Due to the presence of STSMLKA, the retention capacity of nanoparticles in ischemic myocardium was enhanced, while the positively charged SS-31 assisted nanoparticles to detach from lysosomal endocytosis. Compared with free drug, nanoparticles have better targeting effects on ischemic myocardial cells, reducing MI area, inhibiting apoptosis and protecting cardiac function. In addition, clearing excess ROS from oxidative stress sites is an effective strategy for the treatment of myocardial IRI. This finding reveals the benefit of novel H2O2-responsive antioxidant polymer nanoparticles (PVAX) in combating ROS-induced oxidative stress, myocardial IRI, and MI area to improve myocardial function [75]. Hardy and team noted that poly(glycidyl methacrylate) (PGMA) nanoparticles containing curcumin and L-type Ca2+ channel (AID) peptide can effectively reduce oxidative stress and ROS production in myocardial cells and regulate ROS and Ca2 + levels, thereby reducing cardiac IRI [76]. Inhibiting the degradation of ECM may serve as a cardinal mechanism for myocardial ischemia protection. Paramagnetic/fluorescent micellar nanoparticles can target the ECM metalloproteinase inducer EMMPRIN and inhibit matrix metalloproteinase (MMP) activation, further reducing ECM degradation, therefore, EMMPRIN can effectively prevent MMP-mediated ECM degradation to protect the ischemic myocardium [36]. These findings suggest that there are ample strategies to reduce oxidative stress in the targeted ischemic myocardial cells to retard IRI, including ROS-responsive, ROS-sensitive, anti-oxidative stress drugs-loaded and ligand-modified nanoparticles. Furthermore, ECM components can be targeted to inhibit the activation of MMP to prevent IRI and protect cardiac functions.

IRI enhances the permeability of the vascular system and allows nanoparticles to permeate the surrounding tissues. Pioglitazone-loading nanoparticles, which penetrate and accumulate in ischemia-reperfusion myocardium by enhancing vascular permeability, and are absorbed by circulating mononuclear cells through phagocytosis, protect the heart from IRI and cardiac remodeling by antagonizing acute inflammation mediated by mononuclear/macrophages, and promoting cardiac healing after MI. These effects were related to the change of polarity of M1/M2 macrophages in preclinical animal models [77]. Silicon-crosslinked micelles (SCLM) were used as a delivery system for nanoparticles to target the cardiotonic drug Olidone to injured myocardium, successfully maintaining the pumping efficiency of the heart and reducing ventricular remodeling, thereby preventing the positive feedback loop of deterioration of cardiac function; and the myocardial targeting nanoparticles significantly saved the injured heart before irreversible pathological changes occurred [66]. In conclusion, nanoparticles can prolong drug half-life, detect myocardial IRI damage, target ischemic or infarcted myocardium to clear away excess ROS, and utilize enhanced vascular permeability to treat IRI. Various nanoparticle-based approaches for the treatment of IRI are shown in Table 2.

### 2.3. Nanoparticles for Aortic Aneurysm

An aneurysm is a localized dilation of an artery that exceeds 50% of its normal diameter [78]. It upregulates the expression of MMPs, leading to the degradation of elastin and elastin matrix in the arterial wall, resulting in loss of elasticity. Abdominal aortic aneurysms are common among aortic aneurysms, and most of them are found incidentally during physical examination or abdominal ultrasound, CT or MRI. In recent years, many new therapeutic targets for aneurysms have been found, such as MMP, mononuclear/macrophage mediated inflammation, elastin, etc. A drug delivery system designed by Yoshimura and colleagues exhibited the ability to suppress the expression of MMP-9 in mouse aorta [79]. Fukuhara and coworkers produced a pitavastatin conjugate which forms polymeric micelles in water [80] (Table 3). Compared with free pitavastatin, systemic micelle injection can effectively inhibit the expansion of rat abdominal aortic aneurysms by eliminating macrophages and inhibiting MMP-9. The team in Japan also confirmed that nanoparticle-encapsulated pivastatin can improve the pharmacokinetics by promoting the delivery of mononuclear/macrophages to phagocytes and escaping to non-target tissues. Pitavastatin-loaded nanoparticles could inhibit the formation of angiotensin-II-induced abdominal aortic aneurysms via targeting mononuclear/macrophages by offsetting the accumulation of macrophages and matrix degradation, because nanoparticles can correct the vicious cycles that follow a large number of macrophages expressing MCP-1; MCP-1 accelerates mononuclear cell infiltration into vessel walls. This may be the first clinically feasible nanoparticle-mediated treatment of abdominal aortic aneurysm formation [81] (Table 3). This may be a new breakthrough and a more effective strategy in the treatment of aortic aneurysms.

### 2.4. Nanoparticles for Myocarditis

Myocarditis is a disease characterized by myocardial inflammation that increases the risk of dilated cardiomyopathy and heart failure. All patients with suspected myocarditis should undergo clinical evaluation (including laboratory tests), echocardiography, and electrocardiography, while endomyocardial biopsy is required to confirm the diagnosis of myocarditis. Treatment of patients with myocarditis can be divided into general treatment for symptoms (such as heart failure and arrhythmias) and specific treatment for the underlying disease responsible for the etiology of myocarditis, such as immunosuppressive therapy. Macrophage migration is a major histopathological marker of myocarditis and therefore macrophages are potential therapeutic targets for managing the disease. Toita and colleagues [82] emphasize the effectiveness of anti-inflammatory nanomedicine as a treatment option for myocarditis, which may be the first successful report of nanomedicine therapy. In this study, a bioinspired anti-inflammatory nanodrug conjugated with protein G was synthesized, which targets macrophages and induces pole-transition from a pro-inflammatory to an anti-inflammatory phenotype, decreases the levels of pro-inflammatory cytokines, such as IL-1α, IL-6, and TNF-α, while increasing the levels of anti-inflammatory cytokine IL-10. Systemic injection of nanodrug significantly reduced myocardial macrophage migration by 16-fold and myocardial fibrosis by 8-fold in an experimental autoimmune myocarditis mouse model compared to controls (Table 3). This provides a new idea for the effective treatment of myocarditis, and for the treatment of other autoimmune and autoinflammatory diseases.

### 2.5. Nanoparticles for Hypertension

Hypertension is a devastating medical issue endangering human health around the world. Hypertension is diagnosed and classified by measuring blood pressure, while history, physical examination and other tests are helpful in determining the cause and target organ damage. In addition to lifestyle modifications, one or more medications are used to manage the level of hypertension. To date, the greatest contribution of nanotechnology to hypertension is the development of nanomaterials for antihypertensive drugs, such as aliskiren, felodipine, amlodipine, nifedipine, carnidipine, and vasoactive intestinal peptides assembled into nanoparticles for the delivery of antihypertensive drugs [42]. The main advantage of using nanomedicines is that they are able to reduce blood pressure fluctuations by maintaining higher and longer plasma drug concentrations and lower drug doses. He et al. developed a novel transcutaneous drug delivery system (TDDS) based on polyacrylic acid (PAA)-modified MoS2 nanoparticles (PAA-MoS2 NPs) to treat hypertension [20]. In this study, MoS2 NPs were loaded with a β1-adrenergic receptor blocker atenolol (ATE) for treating hypertension. Among them, PAA is a biocompatibility and hydrophilic polymer, which can be used to control water and hydrophobicity properties, and MoS2 NPs are sensitive to near-infrared laser radiation and have good photothermal conversion properties, thereby increasing the release of ATE and promoting ATE penetration via the skin (Table 3). In an earlier study, a single low dose intravenous administration of human VIP-α was found to normalize systemic arterial pressure in spontaneously hypertensive hamsters over an extended period of time, a therapeutically long-acting, biodegradable, and biocompatible peptide nanodrug [83]. Transdermal administration of the invasome of ilaridipine resulted in a 20% reduction in blood pressure elevation compared to control, probably due to the higher permeability of ilaridipine through rat skin in the invaders [84]. Therefore, transdermal nanoparticles can significantly improve the management of hypertension and increase patient compliance, thus providing a new perspective for the management of chronic diseases.

### 2.6. Nanoparticles for PAH

PAH is a progressive fatal disease due to limited circulation to the pulmonary arteries. PAH is characterized by a mean pulmonary artery pressure (mPAP) greater than 25 mmHg, often associated with asthma and chronic obstructive pulmonary disease, among others. Patients with overt exertional dyspnea, albeit with no prior history or signs of PAH, should be suspected with the diagnosis of PAH. Chest X-ray, spirometry, and electrocardiography are performed to eliminate common causes of dyspnea. Doppler echocardiography is performed to assess right ventricular and pulmonary arterial pressure and structural left heart diseases, which are known to evoke secondary PAH. CT or ventilation-perfusion scan can indicate chronic thromboembolic PAH, and arteriography can clearly help to confirm diagnosis. Conventional drug treatments typically involve prostacyclin agonists, endothelin receptor antagonists, and nitric oxide promoters [85], however, the disadvantages of conventional drugs include short half-life, poor stability, high effective dosage, and high risk of systemic side effects [5], thus nanomedicine may be a new therapeutic modality. It was found that polylactic acid–glycolic acid (PLGA) encapsulated sildenafil prolonged the half-life [17]. Metal–organic framework (MOF) nanomedicine of sildenafil can be used to prolong the release and act on blood vessels for a longer period of time for diastole [25] (Table 3). Prostacyclin is an effective vasodilator, and prostacyclin and its analogues can be released by means of lipid carriers in a controlled way. Furthermore, Gupta and team developed a controlled release nanomedicine of fasudil for inhalation with liposomes as nanocarriers, which can produce vasodilatory effects for up to three hours [86]. Another potent vasodilator, such as NO, can cause vasodilation of aorta and lung, but has no effect on pulmonary SMCs. More recently, Mohamed and colleagues developed a new nanoformulation based on a hydrogel-like polymer compound NO-releasing nanoparticles (NO-RP) [21] (Table 3). Release of NO from the NO-RP occurs at a peak at about 120 min, with subsequent continuous release beyond eight hours. Studies confirmed the concentration-dependent diastolic effect of NO-RP on the pulmonary arteries of mice with hypoxic PAH, which in turn could significantly increase the potential of NO for the treatment of PAH. We believe that nanoparticles can significantly enhance the pharmacokinetics of traditional drugs for pulmonary hypertension, prolong the duration of drug action, and improve therapeutic efficacy.

### 2.7. Nanoparticles for Thrombosis

Thrombosis is the leading cause of death in cardiovascular patients worldwide. Abnormal thrombosis can block blood vessels, slow or even stop blood flow, and affect the supply of blood and oxygen in the ischemic area, leading to tissue necrosis and threatened human life. Thrombosis mainly includes arterial thrombosis and venous thrombosis because of different blood flow velocities. Based on the main components of thrombosis, classical antithrombotic drugs mainly encompass antiplatelet drugs, anticoagulants and fibrinolytic drugs. However, the short half-life of these medications, the inability to target and accumulate at thrombus sites, narrowed therapeutic windows, and high incidence of side effects (e.g., severe bleeding) greatly limit their clinical use. Therefore, development of nanotechnology is essential for the treatment of thrombotic diseases. Researchers have explored targeted bionic nanodrug delivery systems based on a variety of endogenous components of thrombotic sites, including platelets, erythrocyte membranes and fibrin, which offer new ideas for efficient thrombolysis and reduction of the risk of bleeding during clinical transformation [87,88,89]. In addition, scientists have used thrombophilia microenvironments (e.g., high levels of oxidation) to trigger thrombolytic drug releases to treat local thrombosis [82]. Physical stimuli, including magnetic and ultrasonic stimuli, have been found to activate nanocarriers to release thrombolytic agents at embolic sites, achieving targeted treatment of thrombosis and significantly improving thrombolytic efficiency [83,84]. Collectively, it is suggested that thrombosis-related nanomedicine can improve the adverse physicochemical properties of drugs, prolong the time of drug circulation in vivo, increase the rapid accumulation of drugs in the target site, and reduce bleeding and other adverse reactions to improve the delivery of thrombolytic drugs. Therefore, thrombosis-related nanotechnology has a good clinical application prospect.

## 3. Challenges and Opportunities of Employment of Nanoparticles in CVD Therapeutics

**Figure 3:**
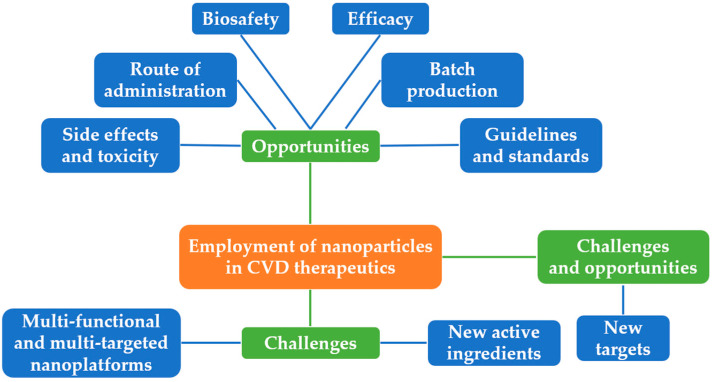
Challenges and opportunities of the employment of nanoparticles in CVD therapeutics.

In recent years, the morbidity and mortality of CVDs remain high, and their harm is increasingly alarming with increasing age. Moreover, the economic burden of CVD will continue to grow, raising the need for novel CVD prevention and treatment strategies. Clinical practice has discovered that classical treatment modalities suffer from many serious insufficiencies and covets. Therefore, cardiovascular nanomedicine appears to represent and surge as one of the hot topics with a broad prospective. Figure 3 illustrates the main challenges and opportunities of employment of nanoparticles in CVD therapeutics. The opportunities are summarized as follows:

(1) New targets: New targets could be identified by continuous ongoing cutting-edge research, which should help to achieve the individualized management of nanoparticles in CVD therapeutics.
(2) Multi-functional and multi-targeted nanoplatforms: Through sustained exploration of nanoparticle synthetic methods, construction of multi-functional and multi-targeted nanoplatforms could be developed to combine drugs with different functions in order to optimize the overall therapeutic outcome.
(3) New active ingredients: With the persistent discovery of active ingredients in traditional Chinese medicine, new active ingredients can be combined with nanotechnology to diagnose and cure CVDs.

Over the past decades, scientists and physicians have gradually realized substantial advances of nanomedicine in improving solubility, release, absorption and bioavailability of poorly soluble drugs, however, challenges remain prior to the full translation of nanotechnology into the clinic.

(1) Side effects and toxicity: A drug delivery system can alter its toxicity and/or induce its own adverse reactions [90] Selenium nanoparticles, for example, have been shown to have anti-atherosclerotic properties in ApoE-/- mice, however, prolonged use of selenium nanoparticles exacerbates oxidative stress and induces disturbances in lipid metabolism [91]. Therefore, careful consideration should be given to the benefit–risk balance when designing and preparing nanomedicines.
(2) Route of administration: Most of the currently designed nanomedicines are intravenously injected. Consequently, this route of administration must be carried out in a hospital setting, which restricts its applicability to the treatment of chronic conditions [92,93].
(3) Biosafety: Nanoparticles are made from organic samples or metallic and synthetic biomaterials. Although the safety and efficacy of nanoparticles have been demonstrated in rodent studies, the safety of nanoparticles has not been systematically studied in large mammalian models of clinical relevance [42]. Secondly, since disease progression and pathology in animal models are significantly different for humans, the same results may not apply to humans, which means that, despite promising preclinical results, nanoparticles have a long way to go before translating into clinical use [41].
(4) Batch production: While good results have been achieved in the formulation of nanomedicine in the lab, there is a limited amount of information about the technology associated with the scale-up of nanomedicines.
(5) Efficacy: Many studies have used different animal models in preclinical studies, making it difficult to compare the results of the various studies derived from different models [41]. One example is the creation of ApoE-/- mouse models in AS studies.
(6) Guidelines and standards: Most of the nanoparticles involved in this study were designed and synthesized by independent research laboratories. There is no consensus with regards to the guidelines or standards for production, characterization and administration of nanoparticles. In this context, it is pertinent to consider the universality and popularization issues for nanoparticles to optimize the advance of the field.

## 4. Conclusions

In summary, ample preclinical studies have indicated the therapeutic potential of nanoparticles in the management of CVDs. In this review, we have emphasized the therapeutic applications of nanoparticles in various CVDs, including ischemic heart disease (AS, angina pectoris, AMI), myocardial IRI, aortic aneurysm, myocarditis, hypertension, PAH and thrombosis. It should be noted that drawbacks and shortcomings are still present for nanotechnology, which warrants future research. Appropriate guidelines and strategies should also be developed to evaluate the risks and therapeutic effects of these drugs to ensure their efficacy and safety in the management of CVDs. At present, there are many studies on the diagnosis and treatment of adult CVDs, yet no relevant studies have been found in children. Therefore, we believe that the research of nanoparticles in Kawasaki disease, child arrhythmia, myocarditis and even related rare diseases (e.g., idiopathic cardiomyopathy, progressive muscular dystrophy, ion channel disease, Pompeii disease) could be a new direction for study, and dosage form change is also a new avenue, for instance, skin patches can increase compliance. Furthermore, nanoparticles need to be applied in clinical trials of cardiovascular therapy more rapidly, efficiently, and economically.

## Figures and Tables

**Table 1:** Different approaches based on nanoparticles for the treatment of ischemic heart disease.

Disease	Structure of Nanomedicine	Nanomaterial	Cargo	Application and Therapeutic Effect	Ref.
AS	Andro-loaded PEG-PPS	PEG-PPS	Andrographolide	Inhibiting the expression of IL-6 and MCP-1, alleviating oxidative stress in macrophages, reducing oxidative stress in the aorta.	[18]
AS	MTX-SPNs	SPN	MTX	Alleviating local and systemic inflammation	[23]
AS	Sim@PMPB NC	PMPB	Simvastatin	Scavenging excess ROS, eliminating oxidative stress, inhibiting oxidized LDL internalization and foam cell formation.	[43]
AS	MM-AT-NPs	MM-NPs	Atorvastatin	Avoiding the clearance of nanoparticles, guiding ROS-responsive nanoparticles to inflammatory tissues, isolating pro-inflammatory cytokines.	[44]
AS	TPCD NP	Tempol, phenylboronic acid pinacol ester	Cyclic polysaccharide β-cyclodextrin	More stable plaque development, fewer cholesterol crystals, smaller necrotic nuclei, thicker fibrous caps, and fewer macrophages.	[45]
AS	HAL@M2Exo	M2Exo	HAL	Targeting and alleviating atherosclerotic inflammation, increasing anti-inflammatory capacity and improving therapeutic efficacy, fluorescence imaging and tracking of AS.	[46]
AS	MM/RAPNPs	PLGA	Rapamycin	Inhibiting the phagocytosis of macrophage, delaying the progression of AS.	[47]
AS	mPEG-PAsp-(g-PEI)-CD36Ab	mPEG-PAsp-(g-PEI)	CD36 antibody-modified siRNA	Reducing CD36 expression, inhibiting upregulation of IL-6 and MCP-1, decreasing the formation of foam cells, and alleviating the pathogenesis of AS.	[48]
AS	GlpNP	PtdSer	Liraglupeptide	Regulatory effect on vascular targets, particularly in the case of SMC inflammation.	[49]
AS	miR-SPION	PEG-SPION	MicroRNA-146a	Downregulating the NF-κB signaling pathway, improving systemic delivery to plaque and AS treatment.	[50]
AS	miR-145 micelle	PAM	miR-145	Promoting the transition of SMCs from synthetic phenotype to contractile phenotype, reducing the size of plaque lesions and necrotic core areas in early and middle atherosclerotic mice, while preserving collagen structure.	[51]
AS	BIN/YCs	Yeast microcapsule	Bindarit	A synergistic effect of modulating plaque lipid levels and inhibiting plaque local inflammation.	[52]
AS	Bifunctional supramolecular nanofiber		IGF-1, naproxen	Regulating cholesterol efflux and inflammation.	[53]
Angina Pectoris	IBH-PNPs	PLGA	Ivabradine Hydrochloride	Increasing the oral bioavailability and half-life.	[15]
Angina Pectoris	Biocomposite polymeric nanofiber	Polymeric nanofibers	Nicorandil	Reducing mucosal ulceration and successfully improving drug bioavailability.	[16]
MI	CTP-EVs-CUR	Extracellular vesicle	Curcumin, miR-144-3p	Enhancing the heart-targeting ability and cardioprotective effect.	[19]
MI	PFP-PFD@NDs-PEG	PFP-NDs-PEG	PFD	Achieving sustained release, inhibiting the conversion of cardiac fibroblasts to cardiac myofibroblasts, and reducing the synthesis and secretion of type I and type III collagen.	[24]
MI	MMNPmiR199a-3p	MMNPs	miR199a-3p	Suppressing hypoxia-induced apoptosis, inflammation and cardiac fibrosis, and promoting cell proliferation.	[54]
MI	TDCNF	SAA1-7	Telmisartan	Reducing apoptosis and inflammation, enhancing antifibrotic potential, and limiting toxicity.	[35]

**Table 2:** Different approaches based on nanoparticles for the treatment of IRI.

Disease	Structure of Nanomedicine	Nanomaterial	Cargo	Application and Therapeutic Effect	Ref.
IRI	OLP@ SCLM	SCLM	Olidone	Maintaining the pumping efficiency of the heart and reducing ventricular remodeling, thereby preventing the positive feedback loop of deterioration of cardiac function.	[66]
IRI	Exenatide/PLL-PEG-PLL	PLL-PEG-PLL	Exenatide	Reducing myocardial IRI by reducing LVEDP and HR, improving LVEF, dp/dtmax and BP.	[73]
IRI	PEG-b-PPS-Rg3	PEG-b-PPS	Rg3	Inhibiting the promotion of oxidative stress, inflammation and fibrosis. Sensitive to cardiac ROS production and has a strong clearance capacity.	[74]
IRI	MCTD-NP	STSMLKA, PLGA	RSV	Better targeting effect on ischemic myocardial cells, reducing MI area, inhibiting apoptosis and protecting cardiac function.	[33]
IRI	NP-C-AID	PGMA	Curcumin, AID peptide	Reducing oxidative stress and ROS production in myocardial cells and regulating ROS and Ca^2 +^ levels.	[76]
IRI	pioglitazone-NPs	PLGA	Pioglitazone	Protecting the heart from IRI and cardiac remodeling by antagonizing acute inflammation mediated by mononuclear/macrophages, and promoting cardiac healing after MI.	[77]

**Table 3:** Different approaches based on nanoparticles for the treatment of aneurysm, myocarditis, hypertension and PAH.

Disease	Structure of Nanomedicine	Nanomaterial	Cargo	Application and Therapeutic Effect	Ref.
Aneurysm	Pitavastatin-Loaded Polymeric Micelle	PEG-PLys	Pitavastatin	Effectively inhibits the expansion of rat abdominal aortic aneurysms by eliminating macrophages and inhibiting MMP-9.	[80]
Aneurysm	Pitavastatin-NP	PLGA	Pitavastatin	Inhibits the formation of angiotensin-II-induced abdominal aortic aneurysms.	[81]
Myocarditis	PSL-G	PSL	Protein G	Targets macrophages and induces pole-transition from a pro-inflammatory to an anti-inflammatory phenotype, decreasing the levels of pro-inflammatory cytokines, while increasing the levels of anti-inflammatory cytokine.	[82]
Hypertension	ATE-loaded PAA-MoS2 NP	PAA-MoS2 NP	ATE	Increases the release of ATE and promotes ATE penetration via the skin.	[20]
PAH	NO-RP-NP	Polymer	NO	Concentration-dependent diastolic effect on the pulmonary arteries of mice with hypoxic PAH.	[14]
PAH	Sil@nanoMIL-89	nanoMIL-89 (MOF)	Sildenafil	Prolongs release and acts on blood vessels for a longer period of time for diastole.	[25]

## Data Availability

The original contributions presented in the study are included in the article material, further inquiries can be directed to the corresponding author.
